# lncRNA DLEU2 modulates cell proliferation and invasion of non-small cell lung cancer by regulating miR-30c-5p/SOX9 axis

**DOI:** 10.18632/aging.102226

**Published:** 2019-09-20

**Authors:** Yongchun Zhou, Hutao Shi, Yaqian Du, Guangqiang Zhao, Xiaoxiong Wang, Quan Li, Junxi Liu, Lianhua Ye, Zhenghai Shen, Yinjin Guo, Yunchao Huang

**Affiliations:** 1 Molecular Diagnostic Center, The Third Affiliated Hospital of Kunming Medical University, Kunming, Yunnan 650118, P.R. China; 2 International Joint Laboratory on High Altitude Regional Cancer, Kunming, Yunnan 650118, China; 3 Kunming Tongren Hospital, Kunming, Yunnan 650118, P.R. China; 4 Department of Cardiothoracic Surgery, The Third Affiliated Hospital of Kunming Medical University, Kunming, Yunnan 650118, P.R. China; 5 Yunnan Key Laboratory of Lung Cancer Research, Kunming, Yunnan 650118, P.R. China

**Keywords:** non-small cell lung cancer, lncRNA DLEU2, miR-30c-5p, SOX9

## Abstract

Increasing evidence indicated that long noncoding RNAs (lncRNA) play critical roles in the progression of multiple cancers and that dysregulation of lncRNA promotes tumor progression. However, the function and underlying mechanism of lncRNA DLEU2 in biological behaviors of NSCLC cells are still largely unknown. Our studies confirmed that lncRNA DLEU2 was highly expressed in NSCLC tissues and cell lines, which was correlated with shorter overall survival in NSCLC patients. *In vitro*, knockdown of lncRNA DLEU2 inhibited proliferation, invasion, migration and induced apoptosis of both A549 and LLC cells; *In vivo*, it suppressed tumor growth and metastasis. lncRNA DLEU2 directly interacted with miR-30c-5p, which further targeted SOX9 and exerted oncogenic functions in NSCLC. Mechanistically, overexpression of lncRNA DLEU2 exhibits tumorigenic effects through downregulating the inhibitory effect of miR-30c-5p on SOX9 expression. In conclusion, Our finding confirmed that lncRNA DLEU2 as a novel oncogenic in NSCLC, which provide a potential novel diagnostic and therapeutic target for NSCLC.

## INTRODUCTION

Lung cancer is the most malignant tumors with the fastest growth rate of morbidity and mortality in the worldwide. In China, the peak of morbidity and mortality has never fallen. According to the statistics, the number of new cases of lung cancer is about 326,600 and the death number due to lung cancer is about 569,400 in 2012 [1]. Previous studies showed that non-small cell lung cancer (NSCLC) accounts for 85% of all lung cancer cases [2]. The biological character of NSCLC is the invasion and metastasis, which is also the first reason for treatment failure and high mortality [3, 4]. Despite a lot of researches have confirmed that multiple tumor-related genes were involved in the regulation of NSCLC progression, and the detail molecular mechanism of NSCLC development and progression are still unclear.

Last past decade, long non-coding RNAs (lncRNAs) have been established as key players in regulating various biological and pathological processes of the tumor. Recently, increasing evidence confirmed that lncRNAs can be involved in many pathophysiological processes of NSCLC, including cell proliferation, migration, invasion, cell cycle processes, and apoptosis [5–8]. For instance, lncRNA DLEU2 is located on chromosome 13q14 [9, 10] and was originally identified as a potential tumor regulator gene. Xie et al. found that overexpression of lncRNA DLEU2 significantly inhibited cell proliferation, invasion, and migration of laryngeal cancer cells [11]. Additionally, dysregulation of lncRNA DLEU2 plays an important role in regulating the growth and metastasis of human cancer, such as pancreatic ductal adenocarcinoma [12], lung adenocarcinoma [13], hematopoietic malignancies [14]. Which indicates that lncRNA DLEU2 may be used as a molecular biomarker for NSCLC diagnosis and treatment. However, the role of lncRNA DLEU2 in the progression of NSCLC was needed to be elucidated further.

microRNA (miRNA) is a class of about 22 nucleotides conserved non-coding small RNA with the regulatory function, acting a pivotal part in the regulation of developmental timing, cell proliferation, and apoptosis as well as tumor development. Among all miRNAs, miR-30c-5p was served as a key regulator gene of multiple malignant tumors progressions, such as NSCLC [15], prostatic cancer [16] and esophageal squamous cell carcinoma [17]. Currently, many studies have manifested that miR-30c-5p, acts as a tumor suppressor gene, was inhibited cell proliferation and invasion through targeting SOX9 in human tumors [18, 19]. Furthermore, the interaction of lncRNA and miRNA play an important role in the occurrence and progression of solid tumor. However, the mechanism of lncRNA DLEU2 promotes NSCLC progression by regulating miR-30c-5p need to be clarified.

In this study, the expression level of lncRNA DLEU2, miR-30c-5p, and SOX9 in NSCLC tissues and cell lines, and the targeted relationship among lncRNA DLEU2, miR-30c-5p and SOX9 were determined, which could regulate the malignant biological behavior of NSCLC cells *in vitro*. Furthermore, the effect of lncRNA DLEU2/miR-30c-5p/SOX9 axis on the growth and metastasis of NSCLC were substantiated *in vivo*. Taken together, our study will provide vital theoretical evidence for explaining the mechanisms of lncRNA DLEU2/miR-30c-5p/SOX9 axis in NSCLC, and at the same time will provide new biomarker and target for the diagnosis and treatment of NSCLC.

## RESULTS

### lncRNA DLEU2 was overexpression in NSCLC patient’s tissues and cell lines

**Figure 1:**
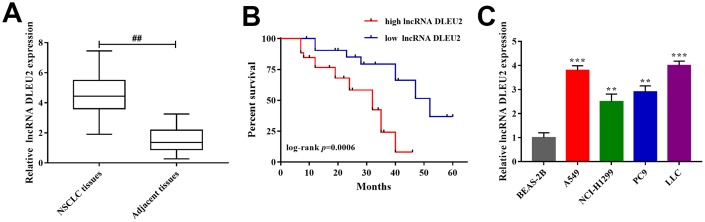
**The expression of lncRNA DLEU2 in NSCLC tissues and cell lines.** (**A**) qRT-PCR was used to detect the expression of lncRNA DLEU2 in NSCLC tissues and adjacent tissues (n=32), ^##^p<0.01, compared with adjacent tissues; (**B**) Kaplan-Meier analyze was applied to evaluate the correlations between lncRNA DLEU2 level and overall survival of NSCLC patients; (**C**) The expression level of lncRNA DLEU2 in NSCLC cell lines by qRT-PCR, ^**^p<0.01, ^***^p<0.001, compared with normal human lung epithelial cells (BEAS-2B).

qRT-PCR was applied to detect lncRNA DLEU2 expression in NSCLC tissues (n=32) and corresponding adjacent tissues. The results showed that the expression level of lncRNA DLEU2 was significantly higher than corresponding adjacent tissues (p<0.01, Figure 1A). 32 paired of cases of NSCLC tissues were divided into groups according to the median expression of lncRNA DLEU2: a high lncRNA DLEU2 expression group (above the median lncRNA DLEU2 expression) and low lncRNA DLEU2 expression group (below the median lncRNA DLEU2 expression). By comparing clinicopathological characteristics, High expression of lncRNA DLEU2 significantly correlated with tumor size, advanced TNM stage (III+IV), and metastasis (p<0.05, p<0.01, Table 1). In addition, the relationship between lncRNA DLEU2 expressions with patient survival was assessed by Kaplan-Meier analysis. The results showed that the high expression of lncRNA DLEU2 remarkably correlated with shorter overall survival compared with lower lncRNA DLEU2 expression in NSCLC patients (p=0.0006, Figure 1B). Furthermore, lncRNA DLEU2 markedly upregulation in NSCLC cell lines compared with human normal lung epithelial cells (BEAS-2B), especially in A549 and LLC cells (p<0.01 or p<0.001, Figure 1C). Thus, these data confirmed that lncRNA DLEU2 was upregulated in NSCLC tissues and cell lines, which was associated with poor survival outcome. Meanwhile, A549 and LLC cells were chosen for subsequent experiments.

**Table 1:** Associations between lncRNA DLEU2 expression and clinicopathological characteristics.

**Clinical feature**	**Number**	**Expression of lncRNA DLEU2**		***p* value**
		**High**	**Low**	
	32	21	11	
Age				p=0.587
≥55	18	12	6
<55	14	9	5
Gender				p=0.545
Male	14	9	5
Female	18	12	6
Tumor size (cm)				*p=0.032
≥5	22	14	8
<5	10	7	3
TNM stage				*p=0.021
I+II	12	4	8	
III+IV	20	14	6	
Metastasis				**p=0.007
Lymphatic	13	9	4
Liver	8	5	3
Bone	6	4	2
No	5	3	2

### Knockdown of lncRNA DLEU2 suppresses cell proliferation, invasion, migration and induce apoptosis of NSCLC cells

**Figure 2:**
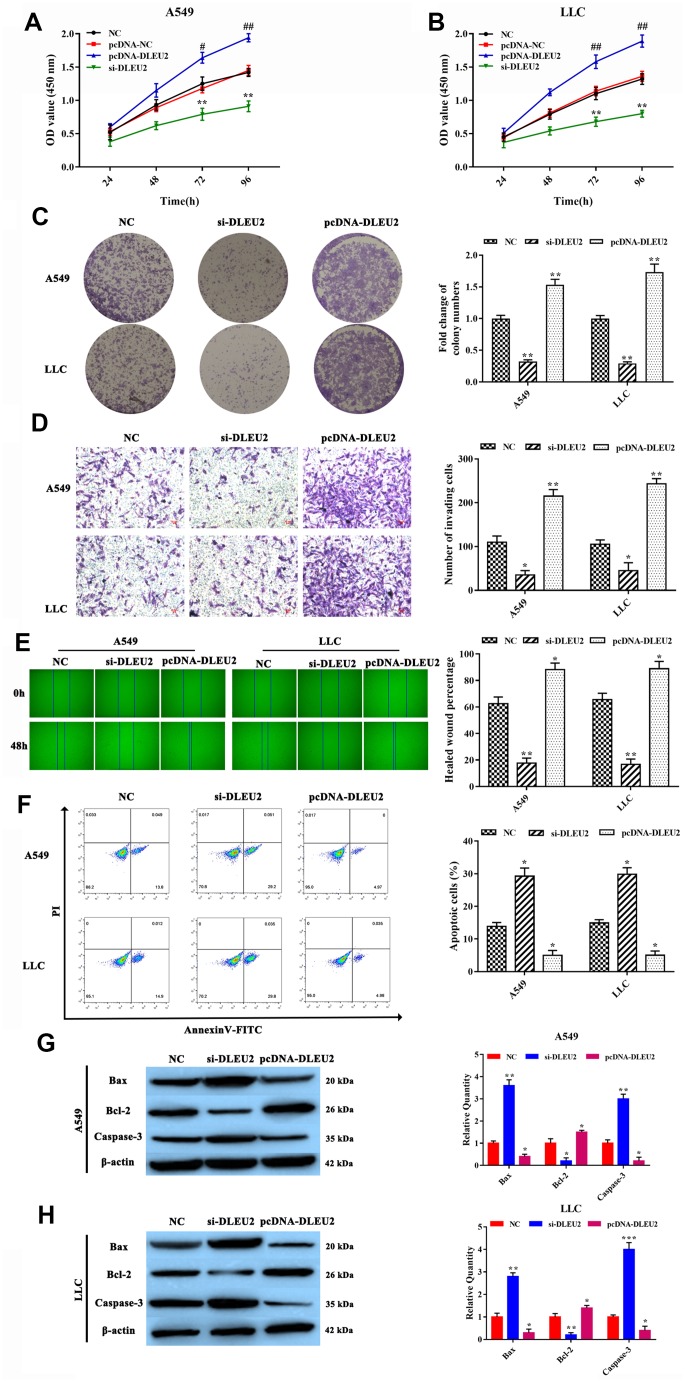
**lncRNA DLEU2 knockdown inhibited cell proliferation, invasion, migration, and induced apoptosis.** (**A**–**B**) Cell viability in transferred with si-DLEU2 and pcDNA-DLEU2 were determined by CCK-8 assay; ^#^p<0.05, ^##^p<0.01, compared with the pcDNA-NC group; (**C**) colony formation of A549 and LLC cells was determined; (**D**) The number of invasion cell was detected by Transwell assay; (**E**) The migration abilities of tumor cells were assessed by wound healing assay; (**F**) The percentage of apoptosis cell was measured by flow cytometry; (**G**–**H**) The expression of apoptosis-related proteins Bax and Caspase-3 were inhibited in si-DLEU2 group by western blotting, while promoted Bcl-2 expression in pcDNA-DLEU2 group compared with NC group. ^*^p<0.05, ^**^p<0.01, compared with NC group.

Based on the expression level of lncRNA DLEU2 in NSCLC tissues and cell lines, we attempted to examine the effect of lncRNA DLEU2 on malignant biological behaviors of both A549 and LLC cells. Primarily, we transfected A549 and LLC cells with three kinds of lncRNA DLEU2-shRNA to verify the knockdown efficiency of lncRNA DLEU2. qRT-PCR showed that lncRNA DLEU2-shRNA^2#^ was the most effective to be chosen for follow-up experiments (p<0.01, Supplementary Figure 1A). Similarly, qRT-PCR was applied to detect the expression of lncRNA DLEU2 in both A549 and LLC cells transfected with pcDNA-DLEU2 (p<0.01, Supplementary Figure 1B). CCK-8 assay results showed that knockdown of lncRNA DLEU2 notably inhibited cell viability in A549 and LLC cells compared with the NC group (both p<0.01, Figure 2A, 2B). Simultaneously, colony formation assay demonstrated that knockdown of lncRNA DLEU2 remarkably inhibited the proliferation capacity of A549 and LLC cells (p<0.01, Figure 2C). Transwell and wound healing assay showed that silencing of lncRNA DLEU2 markedly suppressed the invasion and migration abilities of A549 and LLC cells compared with NC group. (p<0.01, Figure 2D, 2E).

In addition, the Annexin V-FITC/PI double staining assay analyze results showed that lncRNA DLEU2-silenced was contributed to inducing apoptosis of A549 and LLC cells (p<0.01, Figure 2F). Meanwhile, the apoptosis-related proteins Bax, Bcl-2, and Caspase-3 were detected by western blotting, and the same as the result of the Annexin V-FITC/PI double staining analysis (Figure 2G, 2H). Importantly, the upregulation of lncRNA DLEU2 showed a completely opposite result. These results strongly implied that knockdown of lncRNA DLEU2 significantly suppressed cell proliferation, invasion, migration and promoted apoptosis of NSCLC cells.

### miR-30c-5p was a target of lncRNA DLEU2, and miR-30c-5p directly targeted SOX9 in NSCLC cells

**Figure 3:**
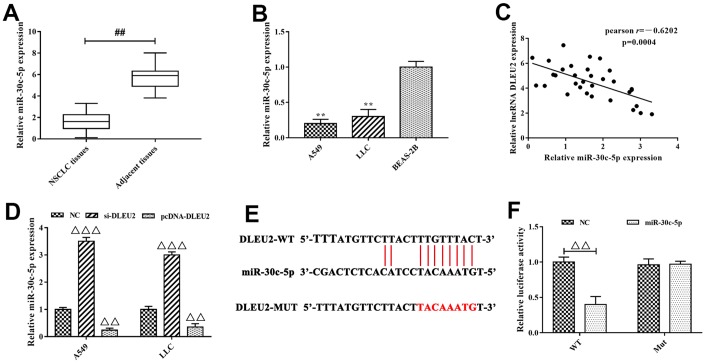
**miR-30c-5p was the target of lncRNA DLEU2.** (**A**–**B**) The expression of miR-30c-5p in NSCLC tissues and cell lines were measured by qRT-PCR, ^##^p<0.01, compared with adjacent tissues; ^**^p<0.01, compared with BEAS-2B cells; (**C**) Spearman’s correlation analysis was used to evaluate the expression relationship between lncRNA DLEU2 and miR-30c-5p; (**D**) The expression of miR-30c-5p in A549 and LLC cells were transferred with si-DLEU2 and pcDNA-DLEU2 by qRT-PCR; (**E**) The bioinformatics analysis result showed that lncRNA DLEU2 had a binding site with miR-30c-5p; (**F**) Dual-luciferase reporter gene assay was used to confirm the target relationship between lncRNA DLEU2 and miR-30c-5p. ^ΔΔ^p<0.01, ^ΔΔΔ^p<0.001, compared with the NC group.

Previous studies confirmed that lncRNA-miRNA-mRNA network plays an important role in the growth and metastasis of cancer including NSCLC [5, 20]. To further understand the mechanism of lncRNA DLEU2 involved in NSCLC progression, bioinformatics tools were applied to analyze the potential interaction between lncRNA DLEU2 and miRNAs. The analysis results showed that miR-30 family (miR-30a-5p, miR-30b-5p, miR-30c-5p, miR-30d-5p and miR-30e-5p) and miR-374 family (miR-374a-5p and miR-374b-5p) were selected to the potential targets of lncRNA DLEU2. These miRNAs expressions were detected by qRT-PCR obtained from NSCLC tissues (Figure 3A and Supplementary Figure 2) and A549 and LLC cells (Figure 3B and Supplementary Figure 3) to investigate the regulatory relationship between lncRNA DLEU2 and miR-30 family and miR-374 family. We detected that miR-30c-5p was the highest expressed one. Meanwhile, there was a significantly negative correlation between lncRNA DLEU2 expression and miR-30c-5p expression in NSCLC tissues by Spearman’s correlation analysis (r=-0.6202, p=0.0004, Figure 3C). Furthermore, knockdown of lncRNA DLEU2 significantly elevated the expression level of miR-30c-5p in A549 and LLC cells by qRT-PCR (p<0.001, Figure 3D), while upregulation of lncRNA DLEU2 markedly decreased miR-30c-5p level (p<0.01, Figure 3D). Taken together, these results indicated that lncRNA DLEU2 may regulate miR-30c-5p expression in NSCLC progression.

Next, to explore whether lncRNA DLEU2 is targeted to miR-30c-5p (Figure 3E), we applied dual-luciferase reporter gene system to establish full-length lncRNA DLEU2 plasmid vector including wild type (WT) and mutant type (MUT) 3’ UTR. The dual-luciferase reporter gene analysis results showed that the luciferase activity of the WT reporter in the lncRNA DLEU2-WT+miR-30c-5p group was significantly decreased compared with lncRNA DLEU2-WT+NC group (p<0.01, Figure 3F), but had no effect on the luciferase activity of the MUT reporter vector. In light of this, we confirm that the target gene of lncRNA DLEU2 was miR-30c-5p, and negatively regulated the expression of miR-30c-5p.

**Figure 4:**
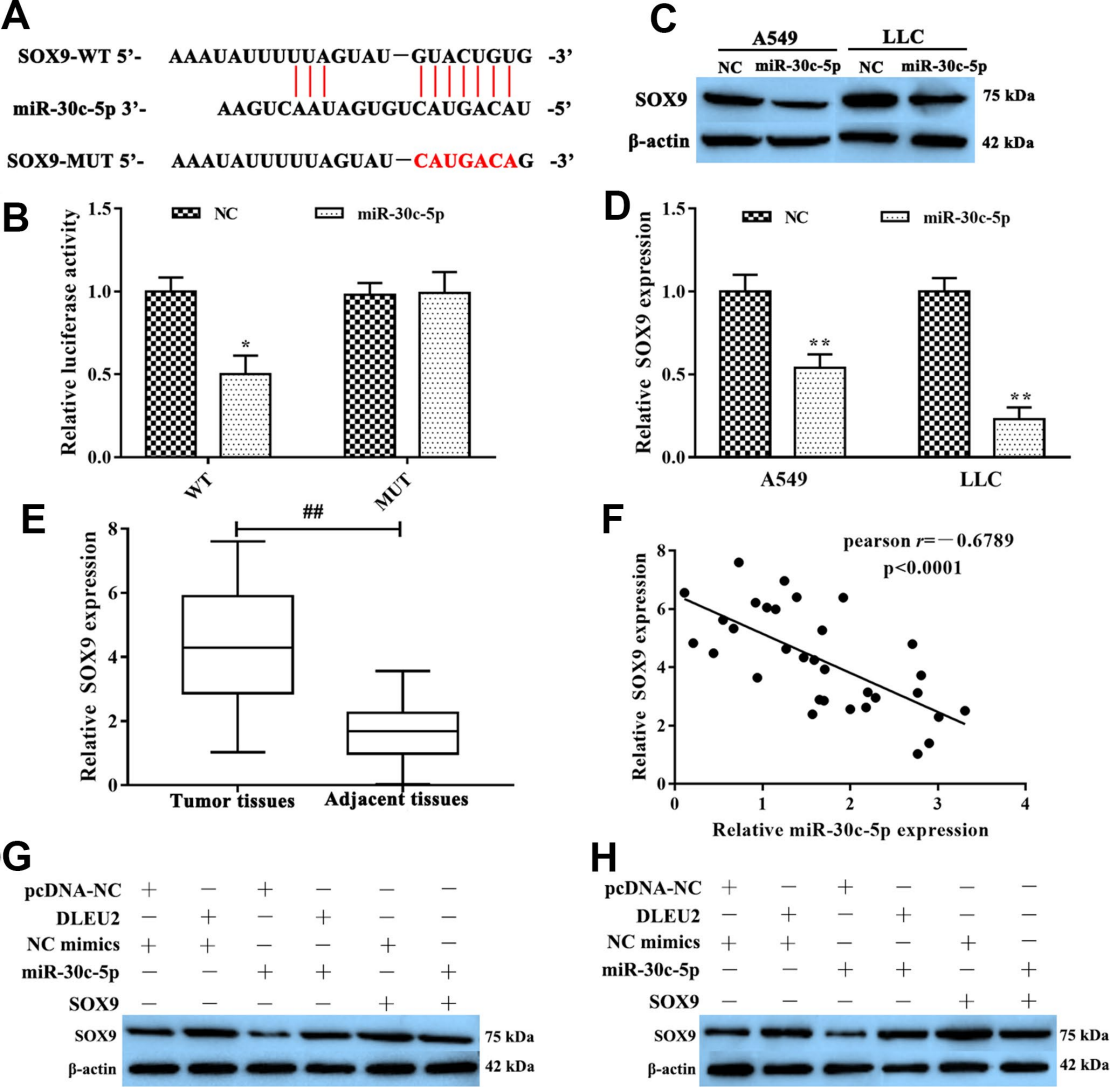
**SOX9 was the target of miR-30c-5p.** (**A**) The bioinformatics analysis result showed that miR-30c-5p had a binding site with SOX9; (**B**) Dual-luciferase reporter gene assay was used to confirm the target relationship between miR-30c-5p and SOX9; (**C**–**D**) The expression of SOX9 mRNA was detected by qRT-PCR and western blotting in A549 and LLC cells which were transferred with miR-30c-5p; (**E**) qRT-PCR was used to determine the expression of SOX9 in NSCLC tissues and adjacent tissues; (**F**) The expression relationship between miR-30c-5p and SOX9 was evaluated by Spearman’s correlation analysis; (**G**–**H**) Western blotting was applied to detect the expression of SOX9 protein in A549 and LLC cells. ^*^p<0.05, ^**^p<0.01, compared with NC group; ^##^p<0.01, compared with adjacent tissues.

Furthermore, we discovered that miR-30c-5p may target at SOX9 directly from the TargetScan database (http://www.targetscan.org/vert_72/) (Figure 4A). To further confirm that miR-30c-5p was specifically binding to the 3’UTR region of SOX9 mRNA to regulate the expression of SOX9 by dual-luciferase reporter gene assay. The results showed that luciferase activity in SOX9-WT+miR-30c-5p mimics group was lower than SOX9-WT+miR-NC group (p<0.05, Figure 4B), but there were no significant between miR-30c-5p mimics or NC were transferred in the SOX9-MUT group. At the same time, we used western blotting to test the expression level of SOX9 when miR-30c-5p mimic was transfected into LLC and A549 cells. The analysis results pointed out that the expression of SOX9 significantly decreased in miR-30c-5p mimics group compared with the NC group (p<0.01, Figure 4C-4D). Additionally, the expression of SOX9 was remarkably higher in NSCLC tissues than adjacent tissues by qRT-PCR (p<0.01, Figure 4E). Furthermore, Spearman’s correlation analysis revealed a remarkably negative correlation between the expression of lncRNA DLEU2 and miR-30c-5p in NSCLC tissues (r=-0.6789, p<0.0001, Figure 4F). In conclusion, these results suggested that SOX9 was the direct target of miR-30c-5p which was negative regulated SOX9 expression.

### lncRNA DLEU2 promotes cell proliferation, invasion, migration and inhibits apoptosis of NSCLC cells via regulating miR-30c-5p/SOX9 axis

**Figure 5:**
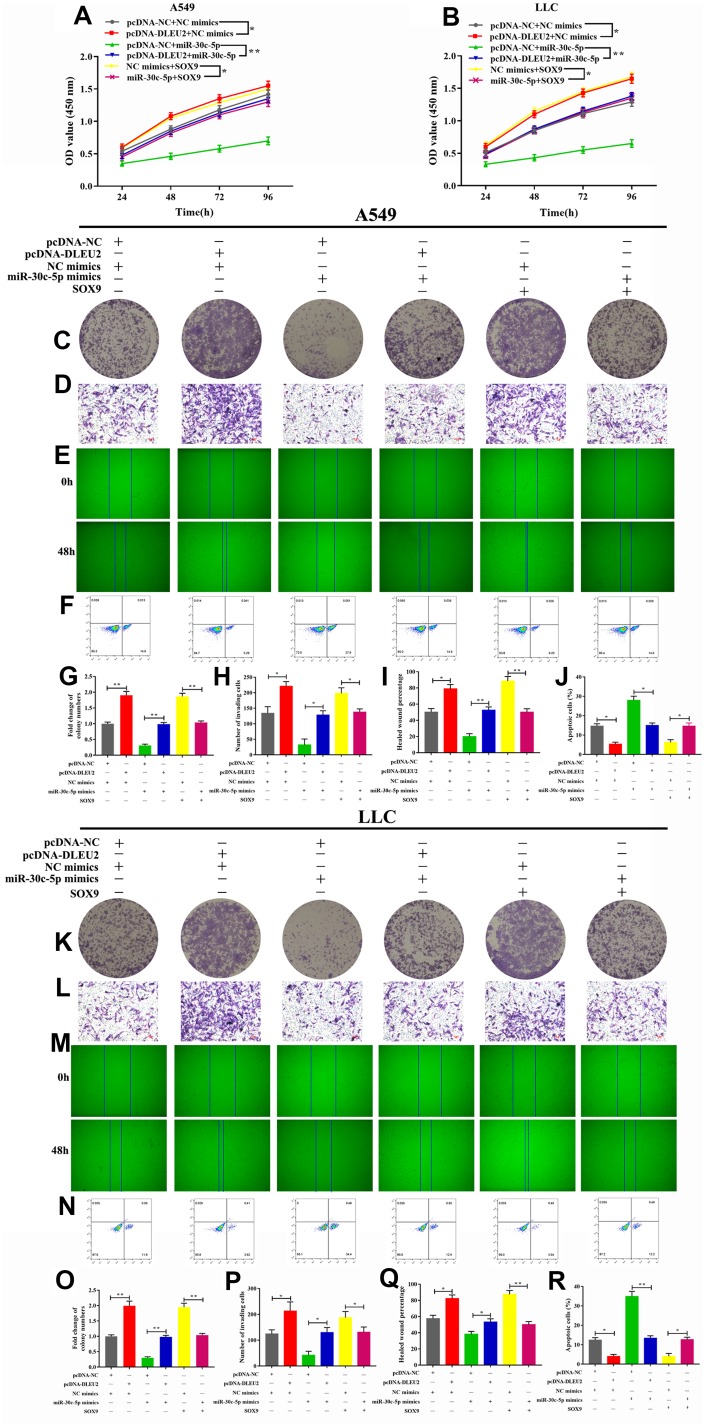
**Effect of lncRNA/miR-30c-5p/SOX9 axis on cell proliferation, invasion, migration, and apoptosis.** (**A**–**B**) Cell viability was determined by CCK-8 assay; (**C**, **G**, **K**, **O**) The colony formatting assay was used to evaluate the proliferation activity of A549 and LLC cells; (**D**, **H**, **L**, **P**) The invasive ability of A549 and LLC cells were detected by Transwell assay; (**E**, **I**, **M**, **Q**) The migration abilities of tumor cells were assessed by wound healing assay; (**F**, **J**, **N**, **R**) Flow cytometric analysis of apoptosis in A549 and LLC cells; ^*^p<0.05, ^**^p<0.01, ^***^p<0.001, compared with control group.

To further determine the molecular mechanism by which lncRNA DLEU2 promotes proliferation, invasion, migration and inhibits apoptosis of A549 and LLC cells by regulating miR-30c-5p/SOX9 axis to promote the development and progression of NSCLC. Western blotting analysis manifested showed that either SOX9 overexpression or lncRNA DLEU2 upregulated resulted in a prominent enhance in SOX9 protein level, and this effect was reversed after co-transfection with miR-30c-5p mimics+pcDNA-DLEU2 or pcDNA-SOX9+miR-30c-5p mimics (Figure 4G, 4H). Moreover, the CCK-8, colony formation, Transwell assay, and wound healing assay showed that overexpression of lncRNA DLEU2 or SOX9 significantly promoted proliferation, invasion, and migration of A549 and LLC cells compared with the negative control group (Figure 5A–5E, 5G–5I, 5K–5M, 5O–5Q). Furthermore, upregulation of lncRNA DLEU2 or SOX9 was contributed to decreasing the percentage of apoptotic cells (Figure 5F, 5J, 5N, and 5R). However, the promotion effect of pcDNA-DLEU2 or pcDNA-SOX9 on the biological behavior of NSCLC cells was reversed by upregulating miR-30c-5p expression. Of note, we transferred miR-30c-5p mimics alone in A549 and LLC cells significantly decreased cell proliferation, invasion, migration and induced apoptosis (Figure 5). Taken together, these results suggested that lncRNA DLEU2 was negative regulated miR-30c-5p to promote proliferation, invasion, migration and induced apoptosis of both A549 and LLC cells by upregulating SOX9 *in vitro*.

### Knockdown of lncRNA DLEU2 suppresses tumor growth and metastasis in vivo

**Figure 6:**
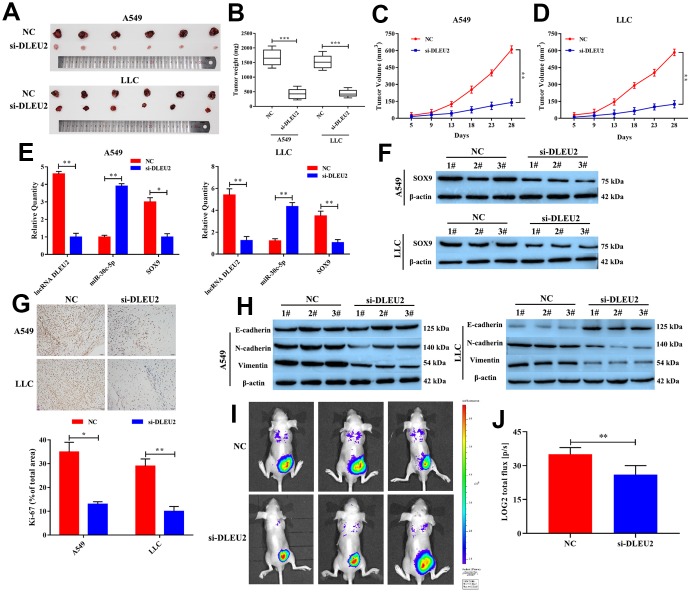
**Knockdown of lncRNA DLEU2 suppresses tumor growth and epithelial to mesenchymal transition in vivo.** (**A**) The tumor size was obtained from nude mice; (**B**) The tumor weight was measured in transferred si-DLEU2 or NC group; (**C**–**D**) The tumor volume curve of nude mice treated with si-DLEU2 or si-NC was analyzed; (**E**) The expression of lncRNA DLEU2, miR-30c-5p and SOX9 were measured by qRT-PCR; (**F**) The expression of SOX9 was measured by western blotting; (**G**) The expression of Ki-67 was detected in tumor tissues by immunohistochemistry; (**H**) Western blot was applied to detect E-cadherin, N-cadherin, and Vimentin protein expression in tumor tissues; (**I**) A549-si-DLEU2 and A549-NC were labeled with firefly luciferase and injected into the abdominal cavity of nude mice (n=3). (**J**) Histogram showing the bioluminescent signal intensity detected using a noninvasive In Vivo Imaging System. ^*^p<0.05, ^**^p<0.01, ^***^p<0.001, compared with NC group.

Based on lncRNA DLEU2 exerts anti-proliferation and pro-apoptotic in both A549 and LLC cells, to further confirm whether lncRNA DLEU2 affect tumor growth and metastasis of NSCLC *in vivo*. As shown in Figure 6A–6D, In lncRNA DLEU2 knockdown group, the tumor volume and tumor weight were significantly reduced compared with the NC group (p<0.01). Moreover, the mRNA expression of lncRNA DLEU2, miR-30c-5p, and SOX9 in NSCLC tumor tissues were determined by qRT-PCR. The results showed that knockdown of lncRNA DLEU2 markedly decreased the mRNA expression of SOX9 and lncRNA DLEU2 compared with the NC group (p<0.01, Figure 6E), while enhanced miR-30c-5p expression (p<0.01). Similarly, western blotting results showed that lncRNA DLEU2 suppression markedly decreased the protein level of SOX9 in tumor tissues compared with the NC group (Figure 6F). Immunohistochemistry results demonstrated that lncRNA DLEU2 knockdown decreased the expression of Ki-67 in xenograft tumor tissues (Figure 6G). Meanwhile, compared with the NC group, silencing of lncRNA DLEU2 significantly suppressed the expression of N-cadherin and Vimentin protein by western blotting, while the expression level of E-cadherin was upregulated (Figure 6H). Furthermore, knockdown of lncRNA DLEU2 significantly suppressed tumor metastasis compared with NC group (Figure 6I). Signals from the group injected with A549-NC cells were significantly stronger than those from the group injected with A549-si-DLEU2 cells (p<0.01, Figure 6J). Therefore, our finding suggests that knockdown of lncRNA DLEU2 may restrict NSCLC growth and metastasis by promoting the expression of miR-30c-5p which downregulation SOX9 *in vivo*.

## DISCUSSION

Multiple malignant tumors are threatening to the safety of our life and will damage our life over a long period of time. In this study, we discussed the molecular biomarker in the development and progression of NSCLC. lncRNA DLEU2 was upregulated in NSCLC tissues and cells, which was enhanced cell proliferation, invasion, migration and reduced apoptosis of A549 and LLC cells by targeting miR-30c-5p. Similarly, overexpression of SOX9 promoted growth and metastasis of NSCLC cells, which was refrained by upregulating miR-30c-5p. Hence, lncRNA DLEU2 promoted NSCLC progression by suppressing miR-30c-5p expression which was dismissed by knockdown of SOX9 expression *in vitro* and *in vivo*.

lncRNA DLEU2 was widely reported to be taken part in regulating cell proliferation, migration, invasion, and apoptosis by targeting tumor-related genes, including miRNA and mRNA. Liu et al. discovered that lncRNA DLEU2-elevated strongly promoted cell viability, migration, and invasion of gastric cancer, which were consistent with our studies results [21]. Moreover, lncRNA DLEU2 was not only involved in NSCLC, but also in clear cell renal cell carcinoma [22] and chronic lymphocytic leukemia [14], which indicated that lncRNA DLEU2 may act as a biomarker for clinical diagnosis of NSCLC. But the mechanisms of potential diagnosis and treatment for NSCLC still needs further study. Perhaps lncRNA DLEU2 was involved in the development of malignant tumors by targeting downstream genes or mediating signaling pathways, but this mechanism is not yet clear.

More and more studies reported that miR-30 family were found to be abnormal in lipid metabolism, adipogenesis, cardiac remodeling and malignant tumors [23, 24]. Herein, miR-30c-5p not only acts as a tumor suppressor gene [25, 26], but also participates in many cellular functions: cell cycle control, DNA damage and repair, gene transcription, cell migration, invasion and apoptosis of human tumor cell by targeting downstream gene [26], including SOX9 [18, 19], Notch1 [27], Runx2 [28] and BCL9 [26]. In addition, the increased expression level of miR-30c was associated with NSCLC poor survival [29]. Meanwhile, Hummel et al. confirmed that miR-30c was contributed to increasing the sensitivity of chemotherapy and radiotherapy in human gastric cancer [30]. Taken together, we speculated that overexpression of miR-30c-5p inhibited the growth and metastasis of NSCLC cells by targeting downstream gene, but the detail mechanisms are not clear and the miR-30c-5p modulated signaling pathway to regulate NSCLC progression will become a study emphasis in further research.

However, there is still some deficiency in this study, such as lacking the mechanism of lncRNA may play a key role in the growth and metastasis of tumor through other pathways. Simultaneously, the effect of lncRNA DLEU2/miR-30c-5p/SOX9 axis on tumorigenesis and metastasis by regulating tumors microenvironment changes remained unclear. Furthermore, the comparatively sophisticated research on the project will be discussed in our further study.

## CONCLUSIONS

In conclusion, our study showed that knockdown of lncRNA DLEU2 could suppress the development and progression of NSCLC through upregulating miR-30c-5p, which inhibited SOX9 expression. In addition, overexpression of lncRNA DLEU2 and SOX9 significantly promoted cell proliferation, invasion and inhibited cell apoptosis by directly downregulation miR-30c-5p *in vitro*. Furthermore, this study hopes to find new molecular targets in the treatment of NSCLC and new biomarkers for diagnosis and prognosis.

## MATERIALS AND METHODS

### Tissue specimen

A total of 32 NSCLC patients (averages age 53±5 years old) tissues and adjacent tissues were collected for this study. This study complies with the ethics committee regulations and conducted with the informed consent of the patients. Meanwhile, these samples were frozen in liquid nitrogen immediately and stored at −80 °C for subsequent experiments.

### Cell culture and transfection

NSCLC cell lines (A549, NCI-H1299, PC9, and LLC) and normal human lung epithelial cells (BEAS-2B) were purchased from the Shanghai Institutes for Biological Sciences of the Chinese Academy of Sciences. These cells were cultured in 10% fetal bovine serum (Hyclone, Logan, USA) and 1% PS (100 units/ml penicillin and 100 mg/ml streptomycin) medium with GlutaMAX (DMEM, Gibco BRL, USA). Cells were habitually passaged every 3-4 days, incubated in an incubator with 5% CO_2_ at 37 °C.

24 h before transfection, A549 and LLC cells were seeded in six-well plates with optimum density and then incubated overnight. pcDNA-lncRNA DLEU2, sh-lncRNA DLEU2, pcDNA-SOX9, miR-30c-5p mimics/ inhibitor and control were transfected into A549 and LLC cells with Lipofectamine 2000 reagent and Opti-MEM medium (Invitrogen Life Technologies) in the light of the specification. The pcDNA-lncRNA DLEU2, sh-lncRNA DLEU2, pcDNA-SOX9, miR-30c-5p mimics/inhibitor and an inhibitor/mimics control (blank plasmid) were bought from Tolo Biotech (Shanghai, China).

### qRT-PCR

Total RNA was isolated from cultured tissues and cells with TRIzol reagent (QIAGEN, Germany) and revere-transcribed into cDNA using PrimeScriptTM RT reagent Kit with gDNA Eraser (Perfect Real Time) (TaKaRa, Japan). qRT-PCR Master Mix (TaKaRa, Japan), the sequence of quantitative PCR primers for miRNA analysis in Table 2. GAPDH, U6 and β-actin as the internal control. The 2-^ΔΔCt^ methods were applied to calculate the relative expression levels of lncRNA DLEU2, miR-30c-5p, and SOX9. The experiment for each group was repeated three times.

**Table 2:** Name and sequences of the primers.

**Name**	**Primer sequences**
lncRNA DLEU2	F: 5′-TCCGAGAGTATAGCGCCACT-3′ R: 5′-ACTGCCCTTTGCTCCAAGTA-3′
GAPDH	F: 5′-CCCACATGGCCTCCAAGGAGTA-3′ R: 5′-GTGTACATGGCAACTGTGAGGAGG-3′
miR-30a-5p	F: 5′-GGGCCTGTAAACATCCTCG-3′ R:5′-GAATACCTCGGACCCTGC-3′
miR-30b-5p	F: 5′-CACCCTGTAAACATCCTACACT-3′ R: 5′-CAGTGCGTGTCGTGGAGT-3′
miR-30c-5p	F: 5′-TGTAAACATCCTACACTCTCAGCAA-3′ R: 5′-GCTGTCAACGATACGCTACGTAACG-3′
miR-30d-5p	F: 5′-TGTAAACATCCCCGACTGGA-3′ R: 5′-GCGAGCACAGAATTAATACGAC-3′
miR-30e-5p	F: 5′-GGCGTGTAAACATCCTCGACTG-3′ R: 5′-GTGCAGGGTCCGAGGT-3′
miR-374a-5p	F: 5′-TTATAATACAACCTGATAAGTG-3′ R: 5′-TGTCAACGATACGCTACGTAA-3′
miR-374b-5p	F: 5′-ATATAATACAACCTGCTAAGTG-3′ R: 5′-GTGCAGGGTCCGAGGTATTC-3′
U6	F: 5′-GGTCGGGCAGGAAAGAGGGC-3′ R: 5′-GCTAATCTTCTCTGTATCGTTCC-3′
SOX9	F: 5′-TGAAGATGACCGACGAGCAGGAGAAG-3′ R: 5′-CTTCCTCGCTCTCCTTCTTCAG-3′
β-actin	F: 5′-CGAGAAGATGACCCAGATCATG-3′ R: 5′-GTGAAGCTGTAGCCGCGCTCGG-3′

F: Forward primer; R: Reverse primer

### Colony formation assay

The 0.25% Trypsin/0.02% EDTA solution was used to digest the A549 and LLC cells at logarithmic phase. The A549 and LLC cells density were 5×10^3^ inoculated in six-well culture plates, and the culture medium was replaced every three days. Subsequently, methanol was used to immobilized cells, when both A549 and LLC cells were incubated for 14 days and stained with 0.5% crystal violet. Finally, Nikon Eclipse E600 microscope (Nikon Instruments, USA) was used to count visible colonies in randomly selected fields. The NSCLC cell clone formation rate was calculated according to the following equation: cell clone formation rate = clone counts/seeded cell counts ×100%.

### CCK-8 assay

Cell Counting Kit-8 (Sigma, Japan) was used to detect the proliferation of NSCLC cells. Cells were seeded in 96-well plates at 5000 cells per well and cultured in 5% CO_2_ at 37 °C incubators for 2 h to adhere cells. Added 10 μL of the cell proliferation reagent CCK-8 to each well and mixed then incubated for 2 h in the incubator. The dual-wavelength microplate reader was used to measure the detection wavelength of 450-490 nm and a reference wavelength of 600-650 nm (Beckman Coulter, USA). Each experiment was set up with three parallel repeats.

### Dual-luciferase reporter gene assay

The cells were seeded in 24-well plates at a density of 60%. according to the manufacturer’s instructions, the reporter construct containing SOX9 wild-type or mutant 3’UTR was co-transfected into cells with miR-30c-5p using Lipofectamine 2000 reagent. After 48 h, the cells were collected and tested for luciferase by dual-luciferase assay system (Promega, USA). The target verification methods of lncRNA DLEU2 and miR-30c-5p is similar to those mentioned above.

### Flow cytometry analysis

Collected the samples mentioned above, washed with PBS, centrifuged at 800 *×g* for 6 min, suspended in ice-cold 70% ethanol/PBS, centrifuged at 800 *×g* for another 6min, and suspended with PBS. Resuspended cells with 100 μL medium and added 5 μL of annexin V and 1 μL of propidium iodide according to the manufacturer’s instructions of Alexa Fluor 488 Annexin V/Dead Cell Apoptosis Kit (Thermo Fisher, USA), and incubated for 15 min at room temperature. BD LSR Ⅱ flow cytometry was used to detect cells apoptosis (BD Biosciences, USA).

### Transwell invasion assay

Matrigel (BD Biosciences, USA) was coated the upper surface of polycarbonate filters. Paced the cells on the surface of the Transwell upper chamber. After 24 h, the invasive cells were fixed with 4% PFA (paraformaldehyde) and rinsed three times with PBS, then stained with 0.1% crystal violet for 10 min and rinsed three times with PBS. Random selection 5 fields of vision for cell count, observation and photography.

### Wound healing assay

NSCLC cells (1×10^6^ cells/well) were treated with different reagents, seeded in six-well plates and cultured until they reached confluence. Wounds were made in the cell monolayer by making a scratch with a 20 μL pipette tip. Plates were washed once with fresh medium after 48 h in culture to remove non-adherent cells. Following this wash, plates were photographed.

### Western blotting

Total protein was extracted for western blotting analysis. The PVDF (polyvinylidene fluoride) was incubated overnight at 4 °C with the primary SOX9 antibody (1:1000 dilution), Bax antibody (1:1000 dilution), Bcl-2 antibody (1:1000 dilution), Caspase-3 antibody (1:1000 dilution), E-cadherin antibody (1:1000 dilution), N-cadherin (1:1000 dilution) and Vimentin (1:1000 dilution), and then with horseradish peroxidase-coupled secondary antibody (1:200 dilution). Signa was detected with chemiluminescence using an ECL kit (Bio-Rad, USA), and the protein bands were quantified by Image J software (National Institutes of Health, USA).

### Nude mice model

Female BALB/c nude mice (4~5 weeks old) were purchased from the Kunming Institute of Zoology, Chinese Academy of Sciences and maintained in a pathogen-free facility. The model was approved by the government's animal ethics committee. A549 or LLC cells (5×10^6^) transfected with NC or si-DLEU2 were suspended in serum-free DMEM medium and then inoculated in left armpit of nude mice (8 mice in each group) at 6-7 weeks old to establish heterotopic transplanted tumor models of NSCLC. Tumor growth was recorded every three days by measuring tumor length and width. After 4 weeks incubation, the mice were killed and the mice tumor tissues to be collected for further evaluated.

### Immunohistochemistry assay

The xenograft tissues were collected and fixed with formalin neutral solution of 10% volume fraction, paraffin-embedded and then sectioned. Subsequently, the DAB horseradish peroxidase color development Kit (Beyotime, China) was applied to conjugated Ki-67 antibody (1:1000 dilution, Abcam, UK) staining at room temperature. The slides were dyed with hematoxylin for 30 seconds, dehydrated and fixed, and then sealed with neutral glue. In addition, all stained images were observed and photographed with a fluorescence microscope (Olympus, Japan) at 400× magnification.

### *In vivo* orthotopic model

Athymic BALB/c mice purchased from the Kunming Institute of Zoology, Chinese Academy of Sciences and maintained in a pathogen-free facility. For experimental metastasis assays, the A549-si-DLEU2 and A549-NC cell lines were labeled with firefly luciferase. Each mouse was then injected with 100 μL of cell suspension (1×10^6^ cells) into the pancreas. Animal health conditions and metastatic progression were monitored. Metastasis was quantified using noninvasive bioluminescence In Vivo Imaging System (IVIS, Xenogen).

### Statistical analysis

The experimental data and image preprocessing were analyzed by SPSS 20 statistical software (IBM, USA) and GraphPad Prism7.0 software (La Jolla, USA), respectively. Besides, student’s t-test was used to analyze the significant difference between the two groups, and the differences between multiple groups were compared by one-way ANOVA. Moreover, P<0.05 was identified as statistically significant.

### Ethics approval

This study was performed after the approval of the Clinical Management Committee of the Affiliated Hospital of Kunming Medical University, with written informed consent obtained from each patient before the operation.

## Supplementary Material

Supplementary Figures

## References

[1] Chen W, Zheng R, Zeng H, Zhang S. The incidence and mortality of major cancers in China, 2012. Chin J Cancer. 2016; 35:73. 10.1186/s40880-016-0137-8 27484217 PMC4971631

[2] Pao W, Chmielecki J. Rational, biologically based treatment of EGFR-mutant non-small-cell lung cancer. Nat Rev Cancer. 2010; 10:760–74. 10.1038/nrc2947 20966921 PMC3072803

[3] Wei L, Sun JJ, Cui YC, Jiang SL, Wang XW, Lv LY, Xie L, Song XR. Twist may be associated with invasion and metastasis of hypoxic NSCLC cells. Tumour Biol. 2016; 37:9979–87. 10.1007/s13277-016-4896-2 26819207

[4] Prabhu VV, Devaraj SN. KAI1/CD82, metastasis suppressor gene as a therapeutic target for non-small-cell lung carcinoma. J Environ Pathol Toxicol Oncol. 2017; 36:269–75. 10.1615/JEnvironPatholToxicolOncol.2017024619 29283339

[5] Chen Y, Lu L, Feng B, Han S, Cui S, Chu X, Chen L, Wang R. Non-coding RNAs as emerging regulators of epithelial to mesenchymal transition in non-small cell lung cancer. Oncotarget. 2017; 8:36787–99. 10.18632/oncotarget.16375 28415568 PMC5482698

[6] Zheng C, Li X, Qian B, Feng N, Gao S, Zhao Y, Zhou B. The lncRNA myocardial infarction associated transcript-centric competing endogenous RNA network in non-small-cell lung cancer. Cancer Manag Res. 2018; 10:1155–62. 10.2147/CMAR.S163395 29795987 PMC5958945

[7] Lu Q, Shan S, Li Y, Zhu D, Jin W, Ren T. Long noncoding RNA SNHG1 promotes non-small cell lung cancer progression by up-regulating MTDH via sponging miR-145-5p. FASEB J. 2018; 32:3957–67. 10.1096/fj.201701237RR 29466052

[8] Jiang H, Zhang H, Hu X, Li W. Knockdown of long non-coding RNA XIST inhibits cell viability and invasion by regulating miR-137/PXN axis in non-small cell lung cancer. Int J Biol Macromol. 2018; 111:623–31. 10.1016/j.ijbiomac.2018.01.022 29337100

[9] Liu Y, Corcoran M, Rasool O, Ivanova G, Ibbotson R, Grandér D, Iyengar A, Baranova A, Kashuba V, Merup M, Wu X, Gardiner A, Mullenbach R, et al. Cloning of two candidate tumor suppressor genes within a 10 kb region on chromosome 13q14, frequently deleted in chronic lymphocytic leukemia. Oncogene. 1997; 15:2463–73. 10.1038/sj.onc.1201643 9395242

[10] Mertens D, Wolf S, Bullinger L, Ohl S, Schaffner C, Döhner H, Stilgenbauer S, Lichter P. BCMSUN, a candidate gene for B-cell chronic lymphocytic leukemia and mantle-cell lymphoma, has an independently expressed homolog on 1p22-p31, BCMSUN-like. Int J Cancer. 2000; 88:692–97. 10.1002/1097-0215(20001201)88:5<692::AID-IJC2>3.0.CO;2-3 11072235

[11] Xie ZZ, Xiao ZC, Song YX, Li W, Tan GL. Long non-coding RNA Dleu2 affects proliferation, migration and invasion ability of laryngeal carcinoma cells through triggering miR-16-1 pathway. Eur Rev Med Pharmacol Sci. 2018; 22:1963–70. 10.26355/eurrev_201804_14723 29687850

[12] Giulietti M, Righetti A, Principato G, Piva F. LncRNA co-expression network analysis reveals novel biomarkers for pancreatic cancer. Carcinogenesis. 2018; 39:1016–25. 10.1093/carcin/bgy069 29796634

[13] Zhu TG, Xiao X, Wei Q, Yue M, Zhang LX. Revealing potential long non-coding RNA biomarkers in lung adenocarcinoma using long non-coding RNA-mediated competitive endogenous RNA network. Braz J Med Biol Res. 2017; 50:e6297. 10.1590/1414-431x20176297 28793054 PMC5572850

[14] Garding A, Bhattacharya N, Claus R, Ruppel M, Tschuch C, Filarsky K, Idler I, Zucknick M, Caudron-Herger M, Oakes C, Fleig V, Keklikoglou I, Allegra D, et al. Epigenetic upregulation of lncRNAs at 13q14.3 in leukemia is linked to the In Cis downregulation of a gene cluster that targets NF-kB. PLoS Genet. 2013; 9:e1003373. 10.1371/journal.pgen.1003373 23593011 PMC3616974

[15] Zhou F, Lu X, Zhang X. Serum miR-30c level predicted cardiotoxicity in non-small cell lung cancer patients treated with bevacizumab. Cardiovasc Toxicol. 2018; 18:284–89. 10.1007/s12012-018-9457-z 29737469

[16] Ostadrahimi S, Fayaz S, Parvizhamidi M, Abedi-Valugerdi M, Hassan M, Kadivar M, Teimoori-Toolabi L, Asgari M, Shahrokh H, Abolhasani M, Mahdian R, Fard-Esfahani P. Downregulation of miR-1266-5P, miR-185-5P and miR-30c-2 in prostatic cancer tissue and cell lines. Oncol Lett. 2018; 15:8157–64. 10.3892/ol.2018.8336 29849810 PMC5962849

[17] Ma T, Zhao Y, Lu Q, Lu Y, Liu Z, Xue T, Shao Y. MicroRNA-30c functions as a tumor suppressor via targeting SNAI1 in esophageal squamous cell carcinoma. Biomed Pharmacother. 2018; 98:680–86. 10.1016/j.biopha.2017.12.095 29304493

[18] Zhang XD, Wang YN, Feng XY, Yang JY, Ge YY, Kong WQ. Biological function of microRNA-30c/SOX9 in pediatric osteosarcoma cell growth and metastasis. Eur Rev Med Pharmacol Sci. 2018; 22:70–78. 10.26355/eurrev_201801_14102 29364496

[19] Liu S, Li X, Zhuang S. MiR-30c impedes glioblastoma cell proliferation and migration by targeting SOX9. Oncol Res. 2019; 27:165–171. 10.3727/096504018X15193506006164 29495977 PMC7848431

[20] Zhu SP, Wang JY, Wang XG, Zhao JP. Long intergenic non-protein coding RNA 00858 functions as a competing endogenous RNA for miR-422a to facilitate the cell growth in non-small cell lung cancer. Aging (Albany NY). 2017; 9:475–86. 10.18632/aging.101171 28177876 PMC5361675

[21] Liu H, Zhang Z, Wu N, Guo H, Zhang H, Fan D, Nie Y, Liu Y. Integrative analysis of dysregulated lncRNA-associated ceRNA network reveals functional lncRNAs in gastric cancer. Genes (Basel). 2018; 9:E303. 10.3390/genes9060303 29912172 PMC6027299

[22] Chen Z, Zhang J, Zhang Z, Feng Z, Wei J, Lu J, Fang Y, Liang Y, Cen J, Pan Y, Huang Y, Zhou F, Chen W, Luo J. The putative tumor suppressor microRNA-30a-5p modulates clear cell renal cell carcinoma aggressiveness through repression of ZEB2. Cell Death Dis. 2017; 8:e2859. 10.1038/cddis.2017.252 28569782 PMC5520909

[23] Irani S, Hussain MM. Role of microRNA-30c in lipid metabolism, adipogenesis, cardiac remodeling and cancer. Curr Opin Lipidol. 2015; 26:139–46. 10.1097/MOL.0000000000000162 25692340

[24] Bertoli G, Cava C, Castiglioni I. MicroRNAs: new biomarkers for diagnosis, prognosis, therapy prediction and therapeutic tools for breast cancer. Theranostics. 2015; 5:1122–43. 10.7150/thno.11543 26199650 PMC4508501

[25] Presneau N, Eskandarpour M, Shemais T, Henderson S, Halai D, Tirabosco R, Flanagan AM. MicroRNA profiling of peripheral nerve sheath tumours identifies miR-29c as a tumour suppressor gene involved in tumour progression. Br J Cancer. 2013; 108:964–72. 10.1038/bjc.2012.518 23175151 PMC3590650

[26] Zhao JJ, Lin J, Zhu D, Wang X, Brooks D, Chen M, Chu ZB, Takada K, Ciccarelli B, Admin S, Tao J, Tai YT, Treon S, et al. miR-30-5p functions as a tumor suppressor and novel therapeutic tool by targeting the oncogenic Wnt/β-catenin/BCL9 pathway. Cancer Res. 2014; 74:1801–13. 10.1158/0008-5472.CAN-13-3311-T 24599134 PMC3959627

[27] Katzerke C, Madan V, Gerloff D, Bräuer-Hartmann D, Hartmann JU, Wurm AA, Müller-Tidow C, Schnittger S, Tenen DG, Niederwieser D, Behre G. Transcription factor C/EBPα-induced microRNA-30c inactivates Notch1 during granulopoiesis and is downregulated in acute myeloid leukemia. Blood. 2013; 122:2433–42. 10.1182/blood-2012-12-472183 23974200 PMC3790511

[28] Zhang Y, Xie RL, Croce CM, Stein JL, Lian JB, van Wijnen AJ, Stein GS. A program of microRNAs controls osteogenic lineage progression by targeting transcription factor Runx2. Proc Natl Acad Sci USA. 2011; 108:9863–68. 10.1073/pnas.1018493108 21628588 PMC3116419

[29] Hu Z, Shu Y, Chen Y, Chen J, Dong J, Liu Y, Pan S, Xu L, Xu J, Wang Y, Dai J, Ma H, Jin G, Shen H. Genetic polymorphisms in the precursor MicroRNA flanking region and non-small cell lung cancer survival. Am J Respir Crit Care Med. 2011; 183:641–48. 10.1164/rccm.201005-0717OC 20889907

[30] Hummel R, Hussey DJ, Haier J. MicroRNAs: predictors and modifiers of chemo- and radiotherapy in different tumour types. Eur J Cancer. 2010; 46:298–311. 10.1016/j.ejca.2009.10.027 19948396

