# Supplementary material for: lncRNA DLEU2 modulates cell proliferation and invasion of non-small cell lung cancer by regulating miR-30c-5p/SOX9 axis
Source: Aging (Albany NY). 2019 Sep 20;11(18):7386–401. doi: 10.18632/aging.102226 (PMC6781974; doi:10.18632/aging.102226)
Supplement: Supplementary Figures [file aging-11-102226-s001.pdf]

SUPPLEMENTARY FIGURES

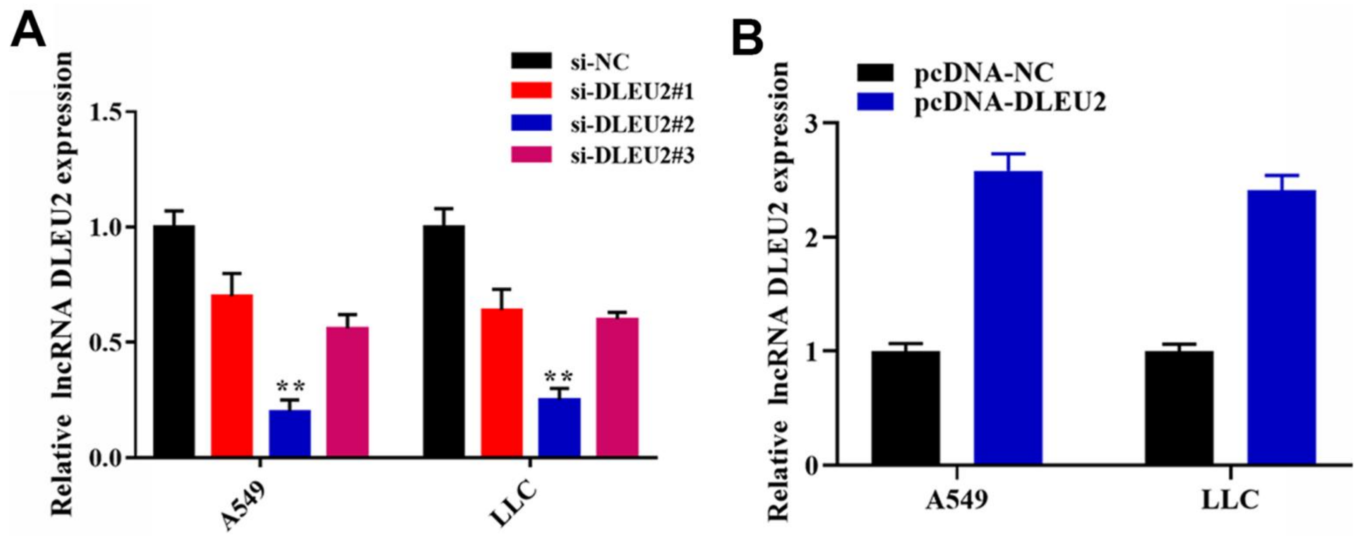

Supplementary Figure 1. Expression of lncRNA DLEU2 in A549 and LLC cells were measured by qRT-PCR.

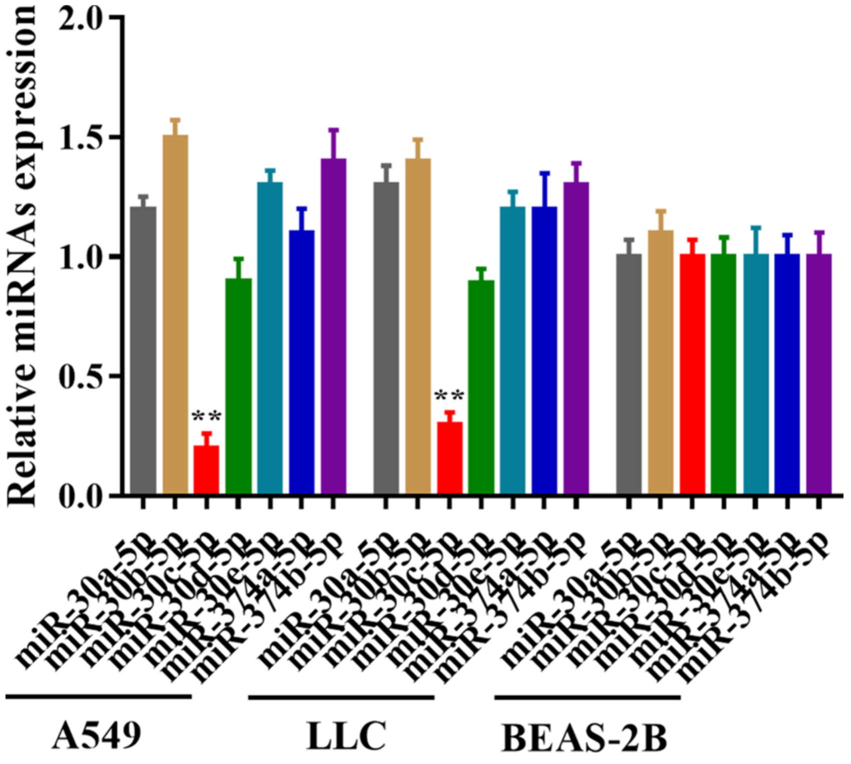

Supplementary Figure 2. Expression of miRNAs in NSCLC tissues was detected by qRT-PCR.

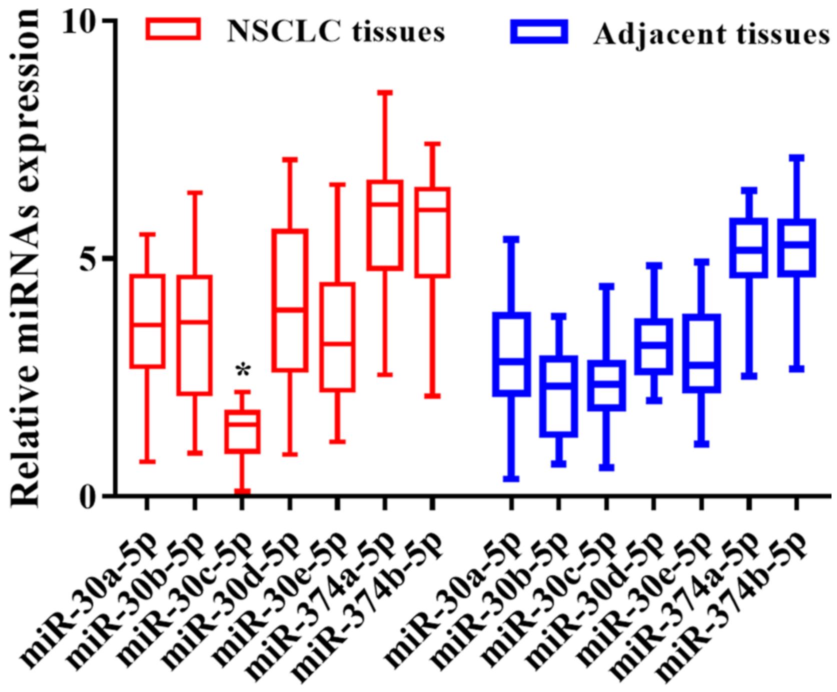

Supplementary Figure 3. Expression of miRNA in A549 and LLC cells were detected by qRT-PCR.
